# Multimodality Intraoperative Neurophysiological Monitoring (IONM) in Anterior Hip Arthroscopic Repair Surgeries

**DOI:** 10.7759/cureus.3346

**Published:** 2018-09-22

**Authors:** Kathryn Overzet, Mark Kazewych, Faisal R Jahangiri

**Affiliations:** 1 Neurophysiology, Axis Neuromonitoring, Richardson, USA; 2 Orthopedic Surgery, Baylor Orthopedic and Spine Hospital, Arlington, USA

**Keywords:** posterior tibial ssep, peroneal ssep, femoral ssep, saphenous nerve ssep, motor evoked potentials tcemep, labral tear repair, hip arthroscopy, train of four, ionm, nerve damage

## Abstract

Arthroscopic hip surgery is performed routinely for the treatment of various hip disorders. Leg traction during labral tear repair, femoroplasty, and acetabuloplasty for hip stabilization can stretch the peripheral nerves. This may cause temporary or permanent nerve injury. This study illustrates the benefit of utilizing multimodality Intraoperative Neurophysiological Monitoring (IONM) during hip surgical procedures.

We performed a retrospective review of 10 arthroscopic hip surgeries with neurophysiological monitoring at one medical center. The patients consisted of six females and four males (mean age: 48.9 years). The procedures were equally divided into left and right-sided procedures. IONM setup included posterior tibial, peroneal, and femoral or saphenous nerve somatosensory evoked potentials (SSEPs), transcranial electrical motor evoked potentials (TCeMEP), train of four (TOF), and electromyography (EMG) from the lower extremities.

All patients exhibited changes in IONM data during the surgical procedure. Changes in the latency and amplitude or loss of the lower SSEPs on the surgical side occurred in 36% of the monitorable SSEPs. The surgeon instructed the team to reduce the leg lengthening by removing traction when changes were observed. The SSEPs exhibited a full recovery in 75% of the affected lower extremity SSEPs. In the two instances of nonrecovery, the SSEP responses remained increased in latency or decreased in amplitude at closing, but the waveform was intact. There were five instances of complete loss of the waveform (four in the ipsilateral leg, and one in the contralateral leg) with recovery after traction was reduced. TCeMEP changes occurred in 53% of the ipsilateral lower muscles monitored. Many of the TCeMEP changes were attributed to ischemia of the feet and could not be resolved intraoperatively.

Multimodality IONM can be a beneficial and protective tool during surgical procedures involving hip and acetabular areas. Early identification of changes in evoked potentials during hip arthroscopy surgeries can minimize post-operative neurological deficits due to peripheral nerve injury and leg ischemia.

## Introduction

Anterior arthroscopic hip surgeries are performed routinely for the treatment of various hip disorders. Femoroacetabular impingement and labral tears may warrant the need for debridement, reshaping, and labral refixation with suture and anchors. To adequately access and repair the joint, the leg is extended away from the rest of the body to create more space between the femur and the acetabulum. The peripheral nerves are at risk of trauma during hip surgeries due to leg traction and may result in temporary or permanent postoperative neurological deficits.

According to Brown et al., the neurological injury is underreported in arthroscopic hip procedures [1]. Peripheral nerves may be directly injured mechanically or thermally, secondary to poor positioning, with leg ischemia or blood clot, and due to stretch from traction to increase the intra-articular space. The patient may also develop a hematoma or inflammatory neuropathy postoperatively [2-8].

A review of the literature reports nerve injury occurring in up to 7.6% of hip surgeries, with revision surgery, excessive leg lengthening greater than 4 cm, hip dysplasia, and the female sex as significant risk factors [1]. The sciatic nerve is the most commonly affected nerve in hip surgery, accounting for 79% of all reported neurological injuries [5, 9]. The peroneal division is particularly susceptible to damage due to its location at the fibular area and smaller size [8]. Post-surgery electromyography (EMG) studies propose that subclinical peroneal nerve injury may be greater than 70% [8].

Intraoperative neurophysiological monitoring (IONM) has been used to assess the functional integrity of the sciatic nerves during hip surgeries since the early 1990s [10]. Somatosensory evoked potential (SSEP) decrease in amplitude or delay in the latency of the lower limb responses is likely due to mechanical or functional loss [10-12].

Anatomy

Obturator Nerve

The obturator nerve is a mixed motor and sensory nerve that arises from the L2-L3-L4 nerve roots. It passes through the posterior region in the iliopsoas muscle and leaves the pelvis via the obturator canal and the superior and lateral parts of the obturator foramen [13]. The motor division supplies the adductor muscles and the sensory division supplies the medial aspect of the thigh. Obturator nerve injury is observed less commonly and does not have as severe of consequences as injury to other peripheral nerves [8].

Superior Gluteal Nerve

The superior gluteal nerve is a mixed motor and sensory nerve that arises from L4-L5-S1 nerve roots. After exiting through the greater sciatic notch, it supplies the gluteal (medius, minimus) and tensor fascia latae muscles [13]. A direct lateral approach to hip surgery puts the superior gluteal nerve at risk [8, 14]. The procedure discussed in this article involves anterior approach hip surgery only.

Sciatic Nerve

The sciatic nerve is a mixed motor and sensory nerve. It originates from the L4-L5-S1-S2-S3 nerve roots. It has two divisions, common peroneal and tibial, which travel in parallel in a single outer sheath [13]. Injury to the sciatic nerve in hip arthroplasty is the most commonly reported neurological injury.

Peroneal Nerve

The common peroneal nerve (L4-S2) splits into two main branches, the deep peroneal and superficial peroneal nerves. It supplies the muscles of the anterior and lateral compartments of the leg. The peroneal nerve innervates the tibialis anterior, extensor hallucis longus, extensor digitorum longus, and peroneus tertius muscles [13]. The peroneal nerve has scarce connective tissues and only a few large funiculi that make it unable to tolerate significant stretch [15].

Tibial Nerve

The tibial nerve (L4-S3) is the larger branch of the sciatic nerve, and it divides into two main branches, the posterior tibial and superficial tibial nerves. It supplies the muscles of the posterior superficial and deep compartments of the leg. The tibial nerve innervates the gastrocnemius, popliteus, flexor digitorum longus, and the flexor hallucis longus muscles [13]. The tibial division has abundant connective tissue and smaller funiculi allowing it to endure more stretch on average than the peroneal division [15].

Femoral Nerve

The femoral nerve is a mixed motor and sensory nerve. It arises from the L2-L3-L4 nerve roots. It passes through the iliopsoas muscle and enters the anterior thigh through the femoral triangle. The motor division supplies the quadriceps muscles. The sensory division supplies the anteromedial thigh and calf regions via the saphenous nerve [13]. Femoral nerve injury does not occur as often as the sciatic injury in hip surgery, however, it is noted to be at risk specifically for the anterior approach [8].

Pudendal Nerve

The pudendal nerve is a mixed motor and sensory nerve. It originates from the sacral plexus, S2-S3-S4 nerve roots, and supplies the anal and urethral sphincter muscles [13]. Pudendal neuropraxia was reported in multiple patients who underwent hip surgery in a study by Contreras et al. [16].

## Materials and methods

Patient history

**Figure 1:**
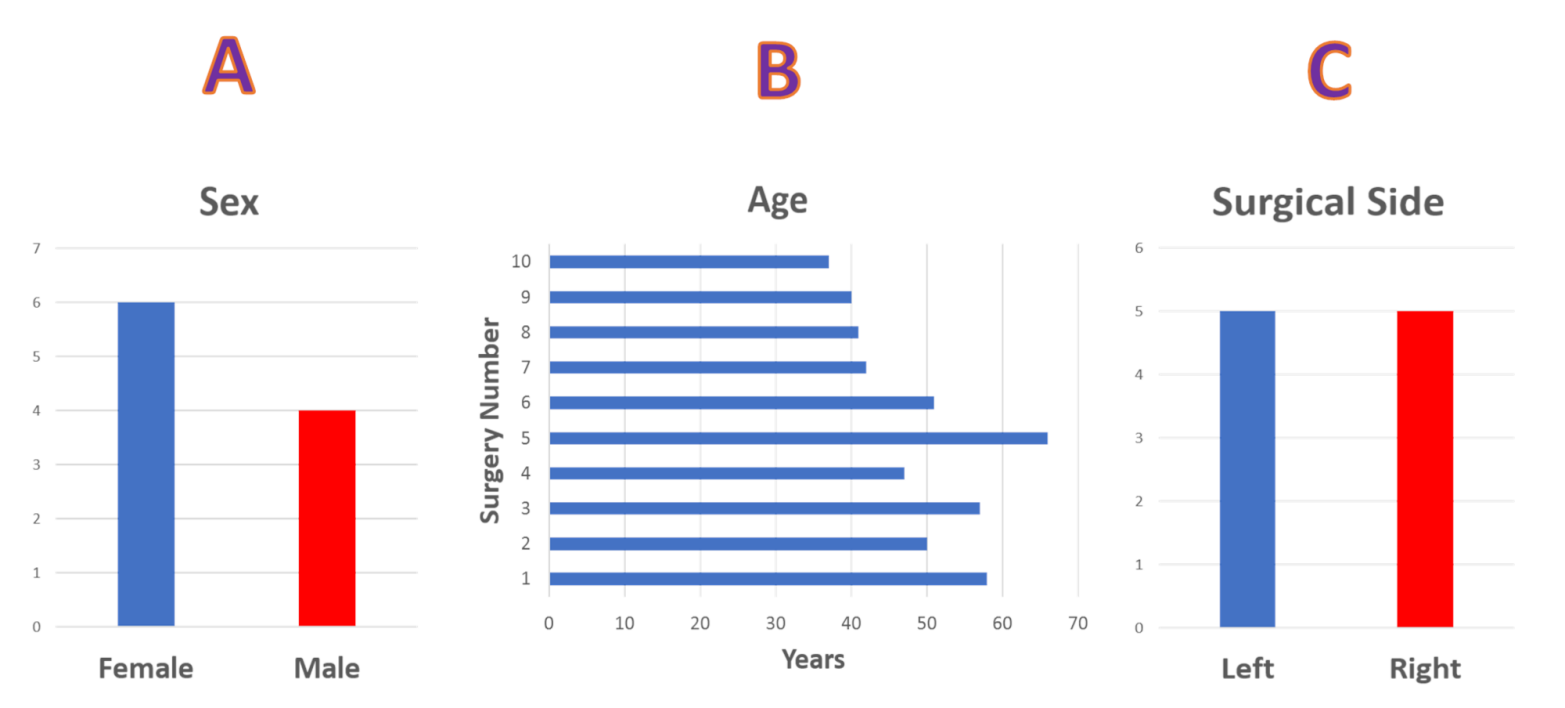
Sex, Age and Operative Side Statistical data showing (A) gender distribution of six females and four male patients. (B) age distribution and (C) surgical side, five left-sided and five right-sided surgical procedures.

We performed a retrospective review of 10 hip neurophysiological monitoring cases at one medical center. The patients consisted of six females and four males with ages ranging from 37 to 66 years (mean: 48.9 years). The procedures were equally divided into left and right-sided procedures (Figure 1).

The primary diagnosis was joint derangement in all cases. Seven of the patients also presented with hip pain, and three of the patients presented with osteoarthritis. All procedures were anterior arthroscopic hip surgeries involving one or more of the following: labral tear repair, femoroplasty, and acetabuloplasty.

Surgical procedure

All 10 patients were positioned supine on a fracture table. Neurophysiological monitoring electrodes were placed after the patient was intubated. The feet were wrapped tightly in elastic bandages and placed in boots that were secured at the end of the bed. A bolster was placed between the legs to keep the patient in place as the leg was pulled and rotated. The contralateral arm was positioned on an arm board, and the ipsilateral arm was positioned across the patient’s chest and secured down. The patient was draped and the exposed skin was prepped.

**Figure 2:**
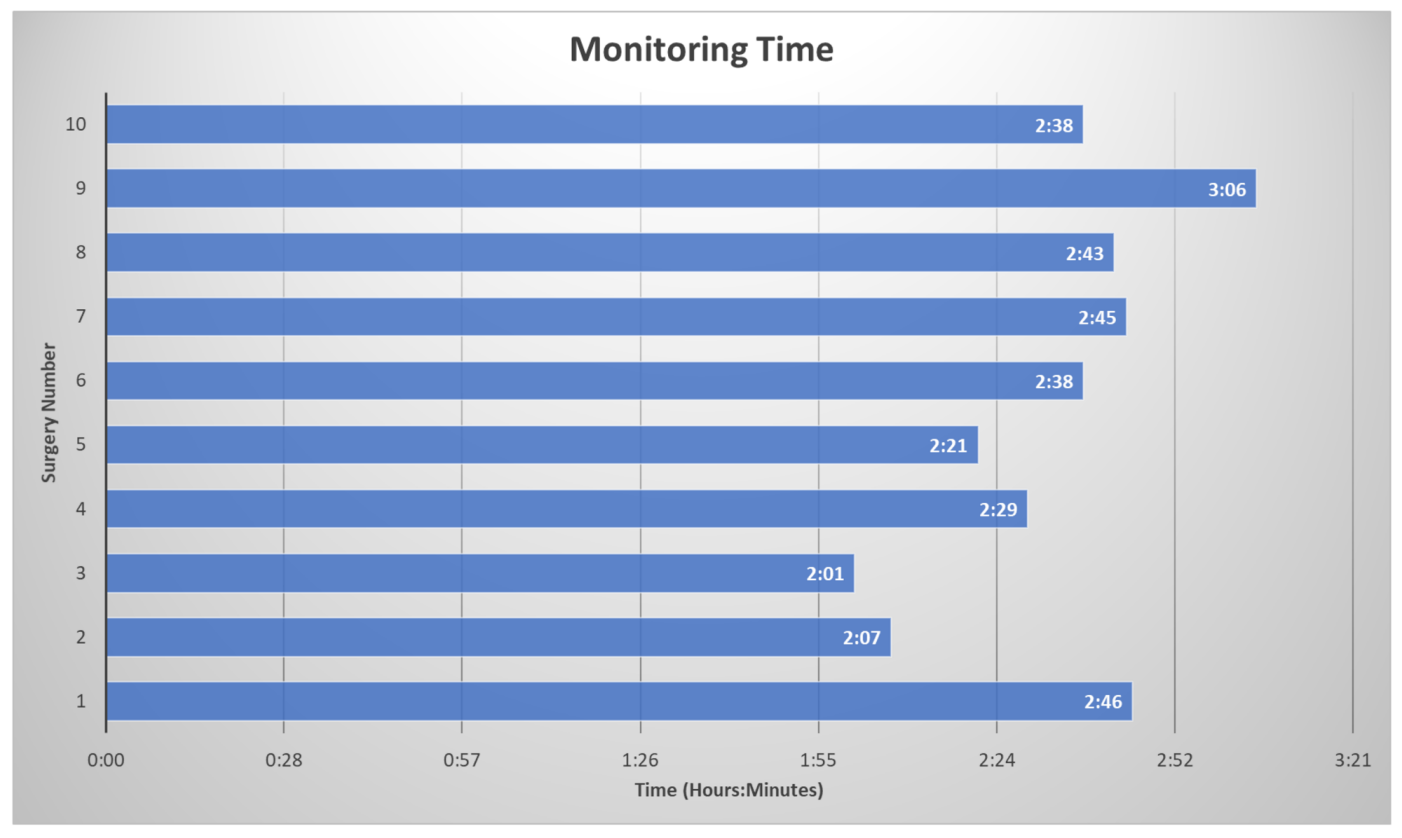
Total Monitoring Time Monitoring time of each surgery from baseline collection to the final trace for each patient in hours and minutes.

The surgeon made small incisions in the skin above the hip joint, minimizing the damage to nearby muscles and other soft tissues. An arthroscope (with a light and a camera lens at its tip) was inserted into one of these incisions to position it in the joint. The live images of the inside of the hip joint were broadcast on a monitor, allowing the surgeon to navigate the joint accurately. The procedure was then performed through another incision. The procedures included debridement, acetabuloplasty, femoroplasty, labral reconstruction, and/or labral repair. The average operating room time was three hours and thirty-one minutes. The average monitoring time was two hours and thirty-three minutes (Figure 2). 

Anesthesia

The anesthetic regimen included both inhalational agents (0.5 to 1.0 mean alveolar concentration desflurane or sevoflurane) and intravenous agents (propofol, remifentanil, ketamine). A short-acting neuromuscular blocking agent was used for intubation in all patients. A train of four(TOF) monitoring technique was utilized by stimulating the posterior tibial nerve and recording from the corresponding abductor hallucis muscles [17]. A TOF of 4/4 was targeted and maintained after intubation.

The surgeon requested a mean arterial pressure (MAP) at 65 mmHg to improve visibility during the repair by minimizing blood flow in the pelvic region.

Intraoperative neurophysiological monitoring (IONM)

**Figure 3:**
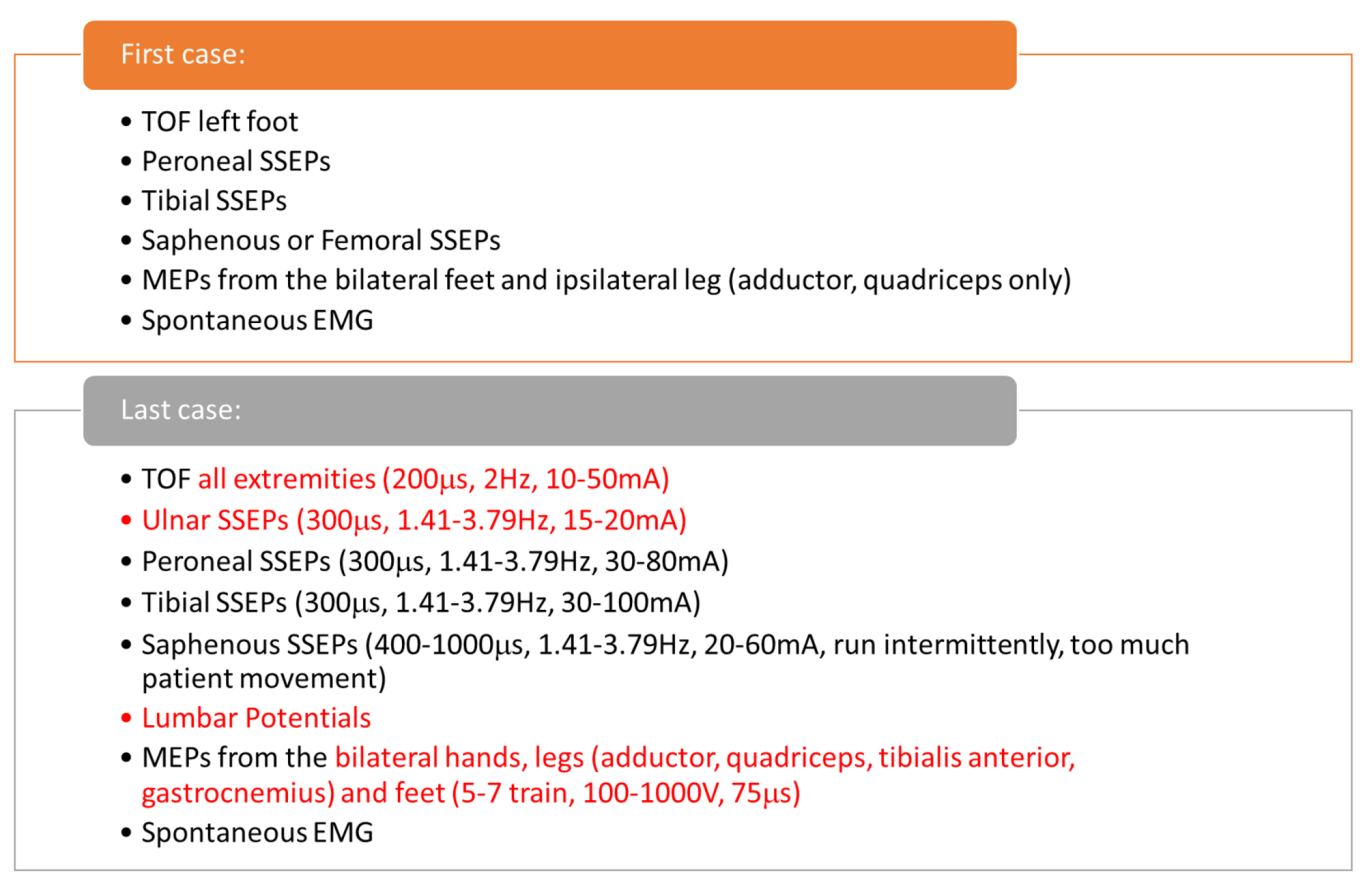
Update in Multimodality Intraoperative Neurophysiological Monitoring (IONM) Protocols Comparison of multimodality monitoring between the first and last surgery. The technique improved over the period of time. TOF: train of four, SSEP: somatosensory evoked potentials, EMG: Electromyography, MEP: motor evoked potentials, mA: milliamperes, µs: microseconds, V: volts, Hz: hertz.

IONM for each procedure varied slightly as the technique was improved and new methods were utilized in the later surgeries (Figure 3). 

Typical monitoring consisted of bilateral SSEPs from the ulnar, peroneal, femoral or saphenous, and posterior tibial nerves, upper and lower transcranial electrical motor evoked potentials (TCeMEPs), spontaneous electromyography (EMG) from the lower extremities, and train of four (TOF) testing [18].

Stimulation surface adhesive electrodes were placed at the wrists for ulnar nerve SSEPs, and at the medial ankles for posterior tibial nerve SSEPs. Peroneal SSEPs were stimulated from either the dorsal surface of the foot or at the fibular head below the knee. Femoral SSEPs were stimulated below the inguinal crease. Saphenous SSEPs were stimulated at the medial thigh. (Stimulation parameters: ulnar: 20 mA, pulse width 300 µsec, repetition rate 2.79 Hz; posterior tibial: 65 mA, pulse width 300 µsec, repetition rate 2.79 Hz; peroneal: 45 mA, pulse width 300 µsec, repetition rate 2.79 Hz, femoral: 55 mA, pulse width 300 µsec, repetition rate 2.79 Hz; saphenous: 55 mA, pulse width 200-1000 µsec, repetition rate 2.79 Hz). Subdermal needle electrodes were placed for SSEP recordings on the scalp according to the international 10-20 system as well as on Cs5 (5th cervical spine vertebra), Erb’s point, and the popliteal fossa. 

Corkscrew or subdermal needle electrodes were placed on the patient’s scalp for TCeMEP stimulation. A train stimulation of 5-9 pulses was utilized (150 to 750 volts, pulse width 50-75 µsec). For TCeMEP and EMG recordings 13-millimeter subdermal needle electrodes were placed in the bilateral abductor pollicis brevis, abductor digiti minimi, tibialis anterior, gastrocnemius, and abductor hallucis muscles. Longer 90-degree hook electrodes (19 mm-25 mm) were placed unilaterally or bilaterally in the adductor and quadriceps muscles.

Impedances were checked and found to be acceptable. Monitoring began, and baselines were obtained. Data for six procedures were recorded by the Cadwell Cascade Pro (Cadwell Industries Inc, Kennewick, WA, USA) and by the Medtronic E4 NIM-Eclipse (Medtronic, Inc., Minneapolis, MN, USA) for the other four procedures.

## Results

All 10 patients exhibited changes in IONM data during the surgical procedure.

**Figure 4:**
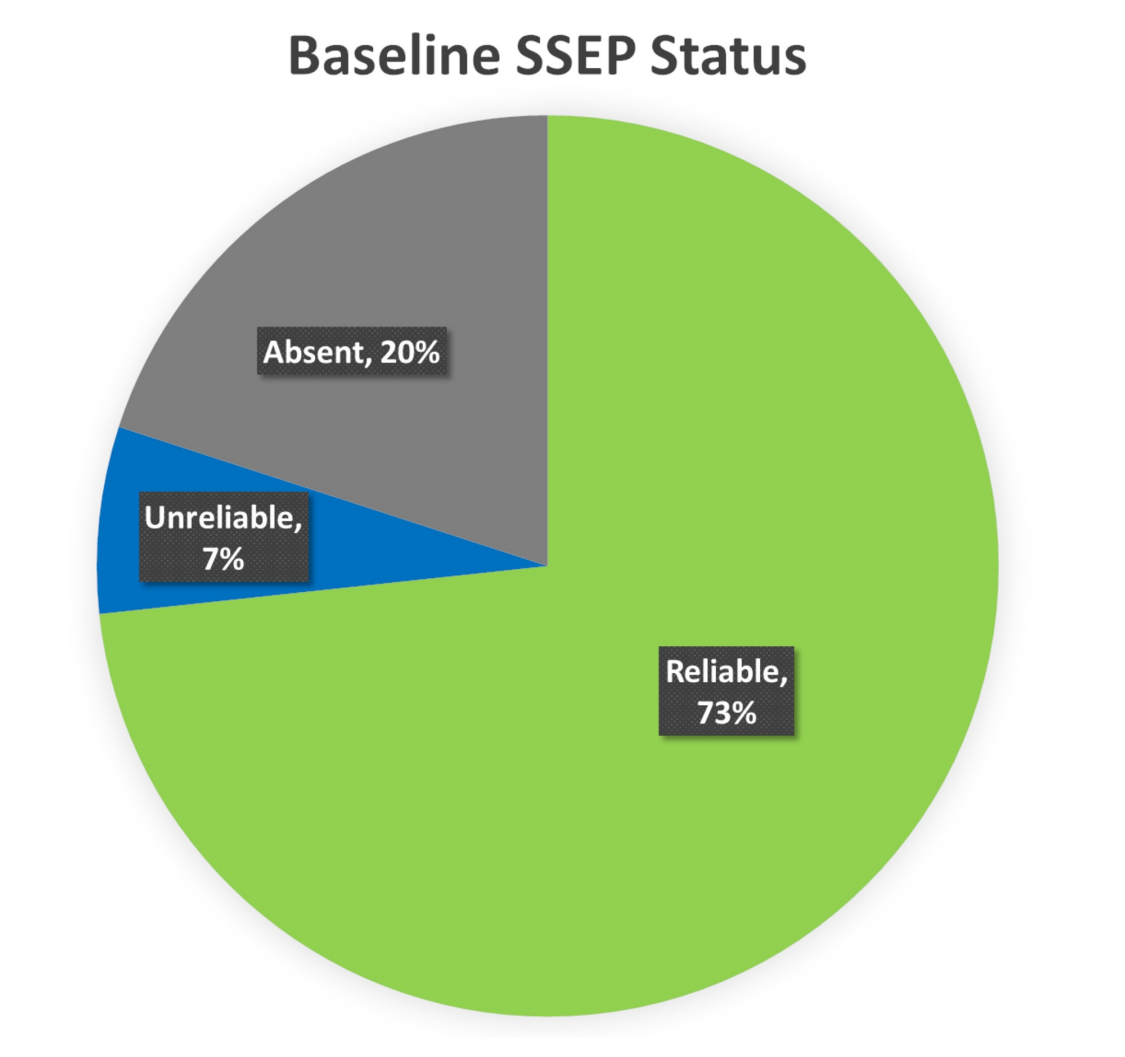
Baseline SSEP Status Ipsilateral baseline lower somatosensory evoked potentials (SSEP) data was reliable in 73% (green), unreliable in 7% (blue), and absent in 20% (gray) of the total nerves monitored.

Somatosensory Evoked Potentials (SSEP):

SSEPs were reliable at baseline in 73% of the lower extremity nerves. The baseline SSEPs were absent in 20% of the lower extremity nerves. In the remaining 7%, the baseline SSEPs were present but exhibited unreliable morphology and reproducibility (Figure 4).

**Figure 5:**
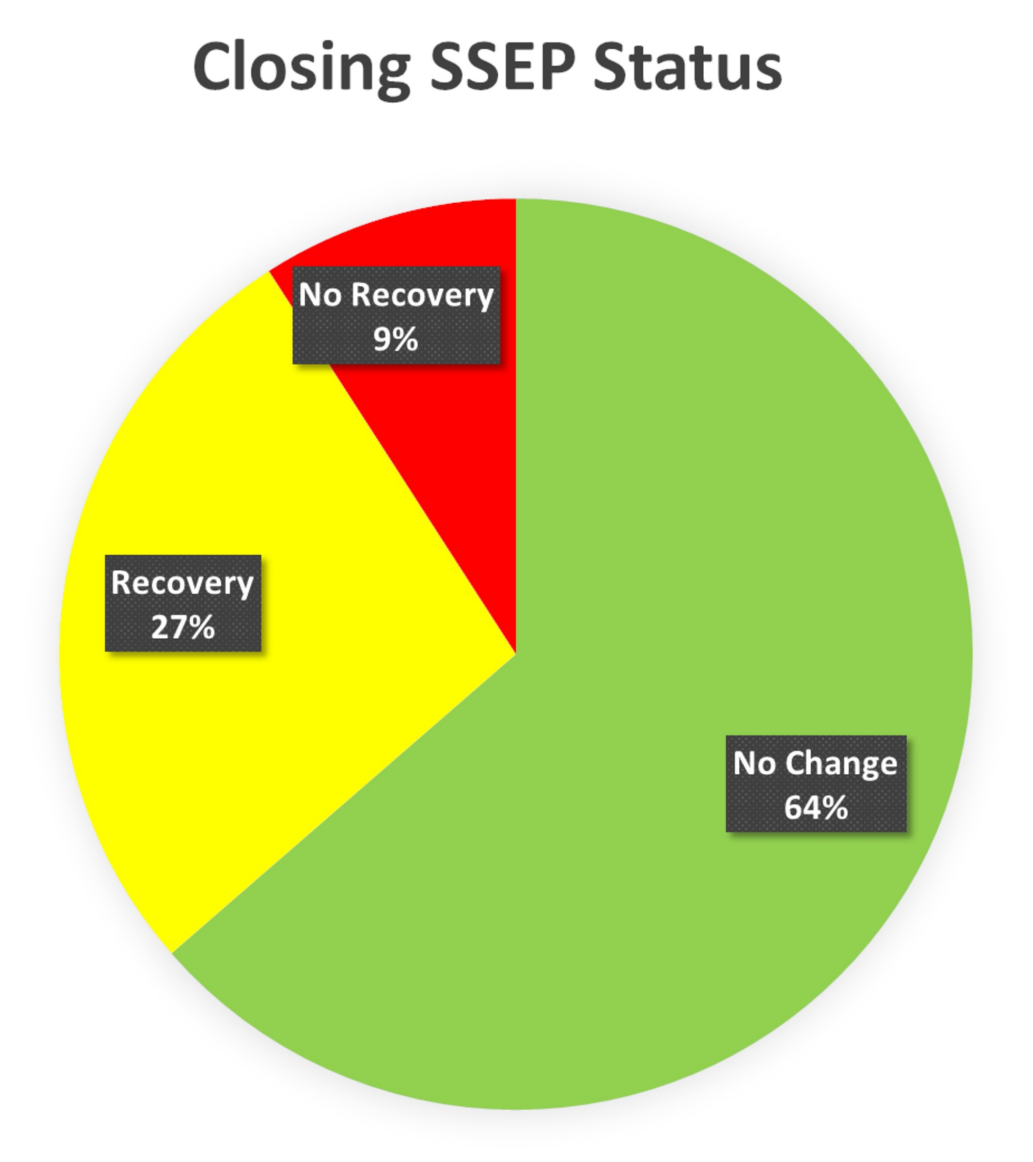
Closing SSEP Status Presentation of status of somatosensory evoked potentials (SSEP) at the end of the surgery (closing). The closing SSEP data was without any change in 64% of the patients, recovered in 27% and had no recovery in 9% of the total monitored SSEP ipsilaterally.

At the end of the surgery, 64% of the monitored lower SSEPs on the surgical side exhibited no significant changes from their respective baselines, while 36% had changes intraoperatively including loss, delay or decrease in amplitude. The SSEPs recovered in 75% of the affected nerves before the end of the surgery. The other 25% remained delayed or decreased in amplitude at closing, but the waveform remained intact (Figure 5).

**Figure 6:**
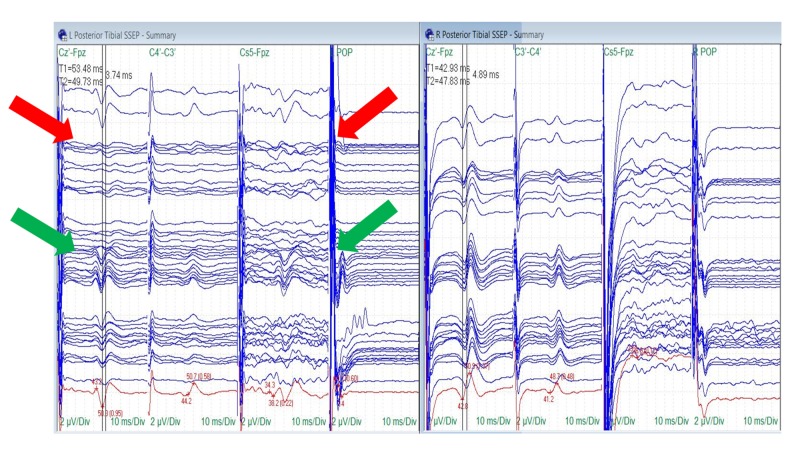
Posterior Tibial Nerve SSEP Stack Loss (red arrows) and recovery (green arrows) of the ipsilateral posterior tibial nerve somatosensory evoked potentials (SSEPs) (cortical, subcortical and popliteal fossa responses) with traction. The surgeon removed turns of traction to allow the SSEPs to recover. Electrode placement according to the international 10-20 system (Cz´: placed at CPz, C3´: placed at CP3, C4´: placed at CP4, Fpz placed at FPz), Cs5: placed at 5th cervical spine, POP: popliteal fossa, µV: microvolts, ms: milliseconds, Div: division.

Nine of the 10 patients had reliable posterior tibial nerve SSEPs at the baseline (90%). Tibial nerve SSEP changes in the ipsilateral side were identified in four of the nine surgeries (44%). In three (75%), there were changes in all recordings (peripheral, subcortical and cortical), including a reduction or loss of the popliteal fossa responses. The popliteal fossa responses remained stable in the fourth surgery (25%) with changes in the subcortical and cortical traces observed only (Figure 6).

**Figure 7:**
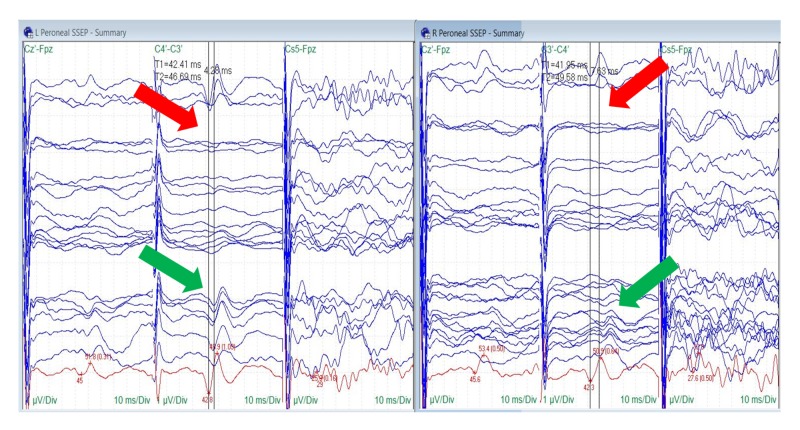
Peroneal Nerve SSEP Stack Loss (red arrows) and recovery (green arrows) of the bilateral peroneal nerve somatosensory evoked potentials (SSEPs). Electrode placement according to the international 10-20 system (Cz´: placed at CPz, C3´: placed at CP3, C4´: placed at CP4, Fpz placed at FPz), Cs5: placed at 5th cervical spine, µV: microvolts, ms: milliseconds, Div: division.

Peroneal nerve SSEPs were reliable at baseline in seven out of 10 patients (70%). Peroneal nerve baseline SSEPs were unreliable in one patient and completely absent in two patients. Changes in peroneal nerve SSEPs were identified in three of seven surgeries (43%). Ipsilateral peroneal changes were observed in two patients (67%), and bilateral changes were recorded in one patient (33%) (Figure 7).

**Figure 8:**
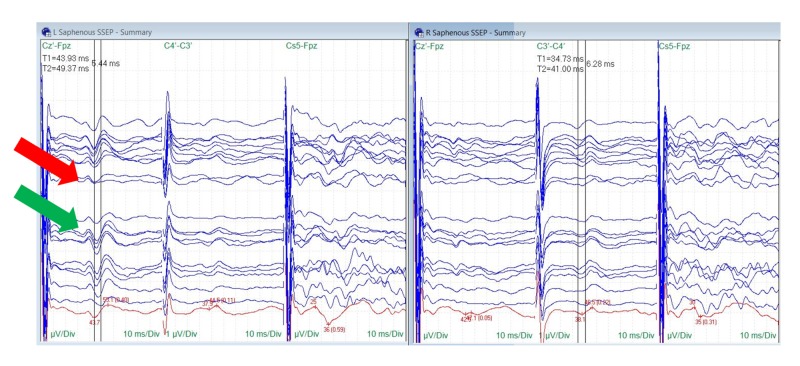
Saphenous Nerve SSEP Stack Temporary amplitude decrease (red arrow) and recovery (green arrow) in ipsilateral saphenous nerve somatosensory evoked potentials (SSEPs) correlating with traction occurred in one surgery, the change occurred comorbidly with peroneal and posterior tibial nerve SSEP changes. Electrode placement according to the international 10-20 system (Cz´: placed at CPz, C3´: placed at CP3, C4´: placed at CP4, Fpz placed at FPz), µV: microvolts, ms: milliseconds, Div: division.

Saphenous nerve SSEPs were reliably monitored in six of the 10 procedures (60%). Baseline saphenous nerve SSEPs were unreliable in one patient and absent in three patients. One of the six procedures with monitorable saphenous signals demonstrated a change (17%). Patient #1 showed a 5-6 millisecond bilateral increase in latency and a temporary decrease in amplitude more prominent on the ipsilateral side. This was accompanied by changes in the ipsilateral peroneal and posterior tibial nerve SSEPs that recovered before the end of the surgical procedure (Figure 8).

The upper extremities were monitored in the last six patients as a control and for positioning changes. Ulnar nerve monitoring was utilized in these patients to detect positioning related changes in the upper extremities. No significant ulnar SSEP changes were observed.

**Figure 9:**
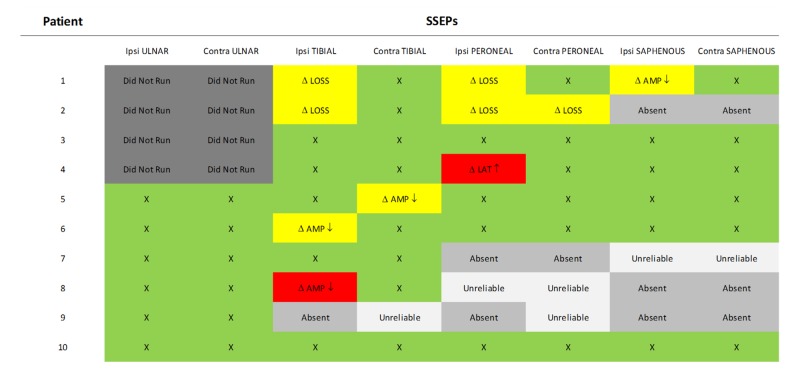
SSEP Data Summary Somatosensory evoked potentials (SSEP) data showing responses that remained stable throughout the procedure and at closing (green), significant changes including loss of the waveform or decrease in amplitude that recovered to values consistent with the baseline prior to closing (yellow), and significant changes including increase in latency or decrease in amplitude that was not resolved prior to closing (red). Ipsi: Ipsilateral, Contra: Contralateral, AMP: Amplitude, LAT: Latency, Δ: Change, X: No change.

One patient (#1) out of 10 had changes in all three lower extremity ipsilateral SSEPs (posterior tibial, peroneal and saphenous nerves) (10%). One patient (#2) exhibited changes in two lower extremity SSEPs (posterior tibial and peroneal nerves) (10%). Three patients (#4, 5, 6 and 8) had a change in only one SSEP (two patients: posterior tibial SSEP, one patient: peroneal SSEP) (30%) (Figure 9).

**Figure 10:**
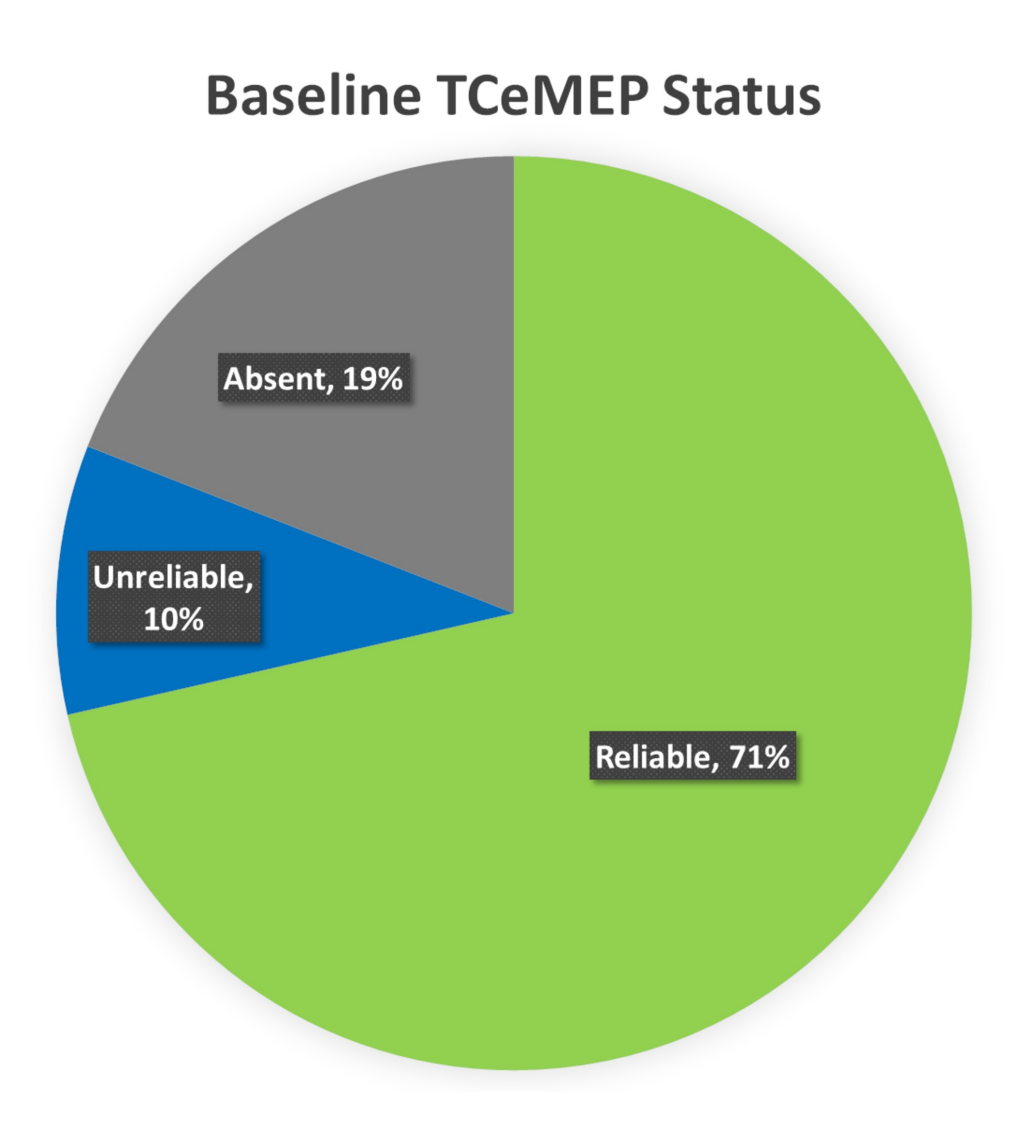
Baseline TCeMEP Status Ipsilateral baseline lower Transcranial Electrical Motor Evoked Potentials (TCeMEP) data was reliable in 71% (green), unreliable in 10% (blue), and absent in 19% (gray) of the total muscles monitored.

Transcranial electrical motor evoked potentials (TCeMEP) and electromyography (EMG):

Lower extremity EMG was monitored in all 10 patients. No abnormal EMG activity was reported in any of the cases. Ipsilateral baseline TCeMEP data was reliable in 71% of the lower extremity muscles. The baseline TCeMEP signals were absent in 19% of the muscles monitored at baseline, and in 10% the baseline TCeMEP signals were unreliable or only intermittently obtained (Figure 10).

**Figure 11:**
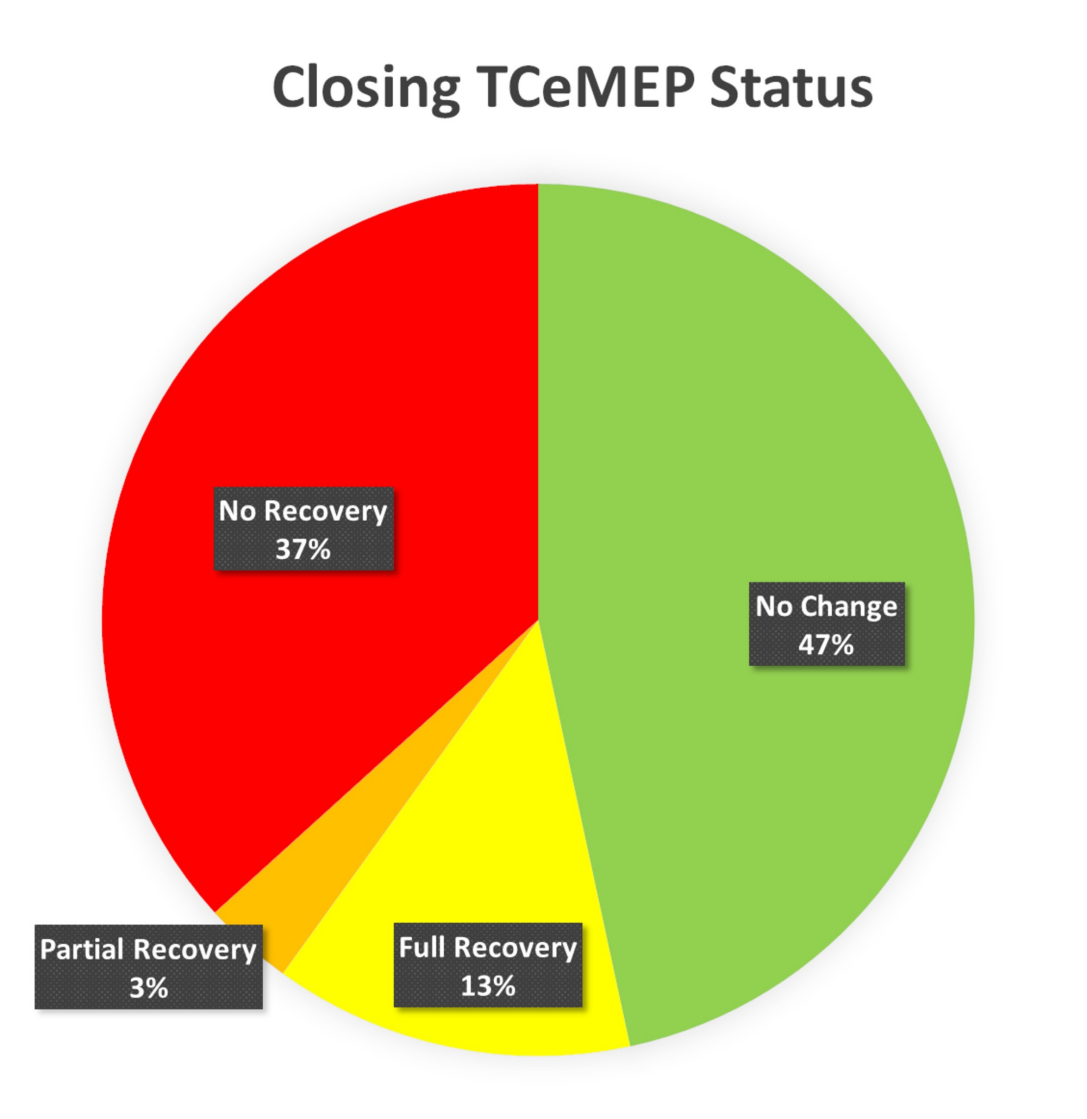
Closing TCeMEP Status Ipsilateral lower transcranial electrical motor evoked potentials (TCeMEP) data was stable throughout the procedure and at closing in 47% (green), fully recovered from significant changes in 13% (yellow), partially recovered in 3% (orange) and exhibited no recovery in 37% (red) of the total muscles monitored.

Of the muscles that were reliably obtained at baseline, 47% remained stable throughout the procedure and at closing. Severe reduction or loss of Compound Muscle Action Potentials (CMAPs) occurred in 53% of the muscles that were reliably monitored. Of the 53% muscles that exhibited changes in CMAPs, only 24.6% made a full response recovery, 5.6% regained a response that was reduced from the baseline at closing, and in 69.8% responses remained absent in lower TCeMEP data (Figure 11).

**Figure 12:**
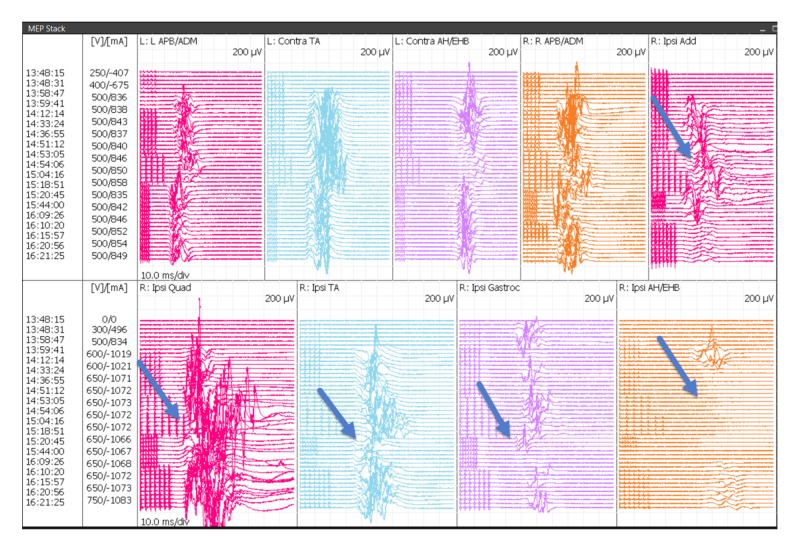
Bilateral Upper and Lower TCeMEP Stack Transcranial Electrical Motor Evoked Potentials (TCeMEP) data showing loss and recovery of motor responses in the ipsilateral adductors, quadriceps, tibialis anterior, gastrocnemius and abductor hallucis muscles (blue arrows). L: left, R: right, Ipsi: ipsilateral, Conta: contralateral, APB: abductor pollicis brevis, ADM: abductor digitiminimi, AH: abductor hallucis, EHB: extensor hallucis brevis, Add: adductor brevis, Quad: quadriceps, TA: tibialis anterior, Gastroc: gastrocnemius, V: volts, µV: microvolts, ms: milliseconds, mA: milliamperes, Div: division.

**Figure 13:**
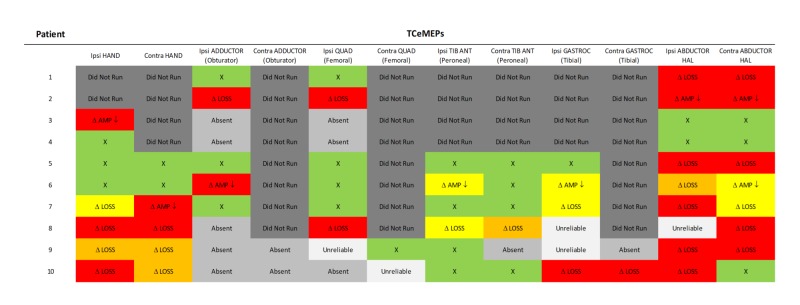
TCeMEP Data Summary Transcranial electrical motor evoked potentials (TCeMEP) data showing responses that remained stable throughout the procedure and at closing (green), significant changes with full recovery prior to closing (yellow), significant changes with partial recovery prior to closing (orange), and significant changes without recovery prior to closing (red). Ipsi: ipsilateral, Contra: contralateral, QUAD: quadricpes, TIB ANT: tibialis anterior, GASTROC: gastrocnemius, HAL: hallucis, AMP: amplitude, LAT: latency, Δ: Change, X: no change.

TCeMEP changes were observed in nine of the 10 surgeries (90%). In these patients, TCeMEP changes were identified in either one muscle (33%), two muscles (33%), three muscles (11%) or four muscles (11%) in the lower extremity. Upper extremity MEPs were monitored in the last consecutive eight surgeries. Unilateral upper extremity TCeMEP changes were observed in one patient (12.5%), while bilateral upper extremity TCeMEP changes were recorded in four out the eight patients (50%) (Figures. 12, 13).

Postoperative

Nine of the 10 patients had no significant postoperative sensory or motor deficits. One female patient had dysesthesia in the feet postoperatively that resolved after two weeks. At the six-month follow-up, all patients continued to have no neurological deficits.

## Discussion

SSEPs were monitored bilaterally and proved to be a consistent indicator of peripheral nerve ischemia and stretch during traction. Changes were quickly identified and reported to the surgeon. SSEP changes were resolved in most cases by reducing the amount of traction. By minimizing the amount of time that the nerve was stretched in patients with sensitivity to traction and ischemia, possible postoperative neurological deficits were avoided. This allowed the surgeon to confidently proceed with traction when significant SSEP changes were not identified, or to reduce the severity or duration of traction when an alert was made.

TCeMEPs were significantly affected and difficult to correlate intraoperatively due to the surgeon’s request for a low MAP hovering around 65 mmHg in most circumstances. Loss or severe amplitude reduction of the foot and hand TCeMEP responses were common and often did not recover prior to the end of monitoring.

Foot TCeMEP changes were observed in eight out of 10 patients (80%). The frequent loss of foot responses, while maintaining leg responses, was likely attributed to the reduced blood flow (requested low MAP) and the tourniquet effect of the elastic bandages and boots on the patient’s feet. Unfortunately, this could not be resolved intraoperatively as the purpose of the bandages and boots is to securely hold the patient’s extremities so that traction is achievable.

Inadequate blood flow to the distal extremities for achieving TCeMEPs was frequent. Hand TCeMEP changes were observed in five out of eight patients (62.5%). Factors such as the patient’s normal blood pressure should be considered when determining what MAP is acceptable for the surgery to maintain adequate blood flow to the distal extremities while still providing joint visibility to the surgeon. TCeMEP responses were more sensitive to MAP changes than SSEP responses. One patient experienced postoperative dysesthesia that may have correlated to inadequate blood flow to the feet.

In a study by Contreras et al., pudendal neuropraxia, neuropraxia of the pudendum, and vulval edema were reported as neurological complications of hip surgery in addition to the more common sciatic neuropraxia and paresthesia of the lower limbs [16]. This is likely due to the pressure created by the bolster between the legs on the fracture bed that holds the patient’s upper body in place as the legs are extended and rotated. Each patient in our study was interviewed for pudendal complications postoperatively by the surgeon. No issues were reported. Future study implications may include pudendal SSEP monitoring, anal sphincter, urethral sphincter TCeMEP monitoring, and Bulbocavernosus Reflex (BCR) monitoring to avoid pudendal nerve injuries.

Injury can occur postoperatively with the development of inflammatory neuropathy. Treatment can improve outcomes with proper diagnosis [19]. Identifying intraoperative changes to nerve function so adjustments can be made during surgery as well as the status of the nerves at the close of the operation is vital information to deliver the best possible outcome. Confirmation of the status of the nerves at closing can help to determine the source and cause of the injury.

A multimodality neurophysiological monitoring during total hip arthroplasty (THA) procedures can be beneficial in reducing postoperative neurological complications due to peripheral nerve injuries. Somatosensory and motor evoked potentials when used independently have different sensitivities and specificities for identification of nerve injury. When used in combination they provide maximum patient protection [20-22]. The utilization of IONM is beneficial to the surgeon with real-time feedback about the patient’s intraoperative neurological status. Immediate and appropriate surgical interventions can prevent permanent nerve damage after an intraoperative alert and provide security to the surgeon to proceed when alerts are not present.

## Conclusions

Arthroscopic hip surgeries in patients carry a very high risk of damaging the sciatic and other lower extremity nerves due to the manipulation and leg traction. In our study, real-time multimodality IONM proved useful for the early identification of peripheral nerve injuries during the surgical procedure. In this study, we had significant changes in sensory and motor evoked potentials in all eight patients. Due to a continuous multimodality neurophysiological monitoring of multiple nerves SSEPs and TCeMEP, the changes were identified. The surgeon was immediately informed about these changes intraoperatively, giving an opportunity to reverse the cause quickly on time. Thus minimizing the duration of nerve compromise and reducing any post-operative neurological deficits. Therefore, to reduce postoperative neurological deficits, we strongly recommend utilizing continuous multimodality neurophysiological monitoring during various hip surgeries.

## References

[1] Brown GD, Swanson EA, Nercessian OA (2008). Neurologic injuries after total hip arthroplasty. Am J Orthop.

[2] Buchholz HW, Noack G (1973). Results of the total hip prosthesis design “St. George”. Clin Orthop Relat Res.

[3] Azansky MGI (1973). Complications revisited the debit side of total hip replacement. Clin Orthop Relat Res.

[4] Amstutuz HC, Ma SM, Jinnah RH, Mai L (1982). Revision of aseptic loose total hip arthroplasties. Clin Orthop Relat Re.

[5] Johanson NA, Pellicci PM, Tsairis P, Salvati EA (1983). Nerve injury in total hip arthroplasty. Clin Orthop Relat Res.

[6] Navarro RA, Schmalzried TP, Amstutz HC, Dorey FJ. (1995). Surgical approach and nerve palsy in total hip arthroplasty. J Arthroplasty.

[7] DeHart MM, Riley LH Jr (1999). Nerve injuries in total hip arthroplasty. J Am Acad Orthop Surg.

[8] Yang IH (2014). Neurovascular injury in hip arthroplasty. Hip Pelvis.

[9] Weber ER, Daube JR, Coventry MB (1976). Peripheral neuropathies associated with total hip arthroplasty. J Bone Joint Surg Am.

[10] Calder HB, Mast J (1995). Sciatic nerve monitoring in acetabular surgeries. Am J EEG Technol.

[11] Farrell CM, Springer BD, Haidukewych GJ, Morrey BF (2005). Motor nerve palsy following primary total hip arthroplasty. J Bone Joint Surg Am.

[12] Jahangiri FR, Al Eissa S, Jahangiri AF, Al-Habib A (2013). Intraoperative neurophysiological monitoring during sacrectomy procedures. Neurodiagn J.

[13] Moore KL, Dalley AF II, Agur AM (2017). Clinically Oriented Anatomy. https://shop.lww.com/Clinically-Oriented-Anatomy/p/9781496347213.

[14] Jacobs LG, Buxton RA (1989). The course of the superior gluteal nerve in the lateral approach to the hip. J Bone Joint Surg Am.

[15] Park JH, Restrepo C, Norton R, Mandel S, Sharkey PF, Parvizi J (2013). Common peroneal nerve palsy following total knee arthroplasty: prognostic factors and course of recovery. J Arthroplasty.

[16] Contreras ME, Hoffmann RB, de Araújo LC, Dani WS, José Berral F (2015). Complications in hip arthroscopy. Rev Bras Ortop.

[17] Ali HH, Utting JE, Gray C ( 1970). Stimulus frequency in the detection of neuromuscular block in humans. Br J Anaesth.

[18] Jahangiri FR (2012). Surgical Neurophysiology, 2nd Edition: A Reference Guide to Intraoperative Neurophysiological Monitoring. https://www.amazon.com/Surgical-Neurophysiology-Intraoperative-Neurophysiological-Monitoring/dp/147516498X.

[19] Laughlin RS, Dyck PJ, Watson JC (2014). Ipsilateral inflammatory neuropathy after hip surgery. Mayo Clin Proc.

[20] Eager M, Shimer A, Jahangiri FR, Shen F, Arlet V (2011). Intraoperative neurophysiological monitoring (IONM): lessons learned from 32 case events in 2069 spine cases. Am J Electroneurodiagnostic Technol.

[21] Laratta JL, Ha A, Shillingford JN (2018). Neuromonitoring in spinal deformity surgery: a multimodality approach. Global Spine J.

[22] Eftekhar Eftekhar, NS NS, Stinchfield Stinchfield, FE FE (1973). Experience with low-friction arthroplasty. a statistical review of early results and complications. Clin Orthop Relat Res.

